# Novel rapid screening device for carotid artery stenosis using video motion analysis: From concept to product

**DOI:** 10.1016/j.csbj.2025.10.055

**Published:** 2025-10-30

**Authors:** Li-Han Lin, Ching-Chang Huang, Peng-Jie Wang, Hsien-Li Kao, Hsin-Fang Hsu, Yu-Ting Shen, Ko-Wei Fan, Hao-Ming Hsiao

**Affiliations:** a Department of Mechanical Engineering, National Taiwan University, Taipei, Taiwan; b Division of Cardiology, Department of Internal Medicine and Cardiovascular Center, National Taiwan University Hospital, Taipei, Taiwan; c Department of Computer Science and Information Engineering, National Taiwan University, Taipei, Taiwan

**Keywords:** Ischemic Stroke, Carotid Artery Stenosis, Video Motion Analysis, PulStroke, Rapid Screening Device

## Abstract

Stroke is the leading cause of death worldwide. Carotid artery stenosis (CAS) is an early indicator of stroke, as well as a risk factor for vascular diseases. Currently, the preferred choice among non-invasive diagnosis tools for assessing CAS severity is carotid Doppler ultrasound; however, it is impractical for use in screening. In this study, the PulStroke project was initiated in our laboratory to transform the video motion analysis (VMA) developed by the authors into a commercial product using a user-centric design approach, with the goal of completing CAS screening with ease. A user takes a 20-second video clip with the PulStroke device, and with just one simple screen touch, the video clip is recorded and automatically uploaded to a cloud server for the assessment of CAS risk. VMA analyzes the video clip, and a risk report is sent to the user’s account in 5 min. This process is achieved with an automated system featuring hardware integration and streamlined operation flow via backend/frontend software architecture. This paper presents the methodology used to advance the technology readiness level from 1 to 6, highlighting the evolution of the device and method, called the PulStroke as a potential brand name, from concept to product. As part of the technology maturity evaluation, the PulStroke was tested on target users, i.e., medical and non-medical professionals, for the user experience and post-screening feedback. Most users found the PulStroke to be fully functional and easy to use, and the satisfaction rate was higher than 88 %. The PulStroke device could be incorporated into future clinical practice to provide the general public with quick and cost-effective CAS screening for the first time.

## Graphical Abstract

**Figure:**
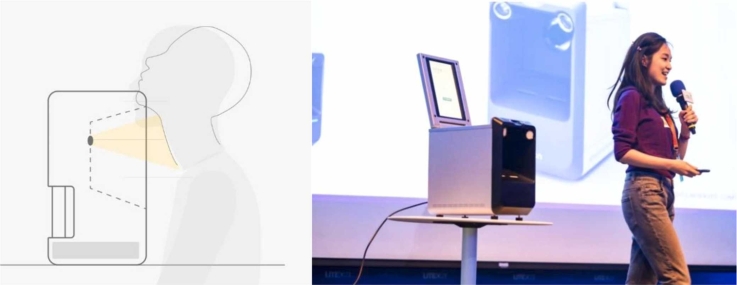

## 1 Introduction

Stroke is the leading cause of death and a major cause of disability worldwide. The long-term disabilities of stroke patients put significant burdens on individuals, families, and even societies [1]. Two early indicators for stroke are carotid artery stenosis (CAS) and atrial fibrillation (AF) [2]. According to the British Heart Foundation, CAS is a major cause of stroke, accounting for about 20 in 100 of all cases [3].

Carotid artery stenosis occurs when a build-up of fatty deposits, called plaque, blocks the carotid arteries on either side of the neck that supply blood to the brain. Currently, carotid Doppler ultrasound is the preferred choice for diagnostic testing due to its reasonable sensitivity and specificity [4]. However, even the most modern, portable version of this tool still requires professional medical staff to operate it, and this requirement creates resource limitations and operator variability. Two other techniques, computed tomography angiography (CTA) and magnetic resonance angiography (MRA), are accurate, but they are expensive and impractical for screening purposes. Therefore, CAS screening with novel tools for early detection and timely intervention is a potential unmet need for asymptomatic patients, or even the general public, to reduce stroke incidence and mitigate the severity of disabilities.

Smart medical devices, such as wearable health devices, are an emerging technology that enables continuous monitoring of human vital signs such as heart rate, respiratory rate, blood pressure, and oxygen saturation in daily life [5]. Some devices can even track more advanced physiological parameters by incorporating the electrocardiogram (ECG), photoplethysmogram (PPG), and impedance cardiogram (ICG). These devices can improve health outcomes and be used as diagnostic tools in the early detection of abnormal physiological parameters or adverse events [6]. Although these wearable devices provide acceptable sensing outputs, they may suffer from signal loss due to improper contact of the sensors with the skin. Therefore, in recent years, there has been a growing demand for and rapid progress on contactless technologies, particularly visual contactless physiological monitoring (VCPM), which uses videos recorded by cameras to monitor vital signs [7]. Although VCPM is new and presents numerous challenges, it has the potential to revolutionize digital health and telemedicine in future clinical practice. In fact, studies focusing on vital sign detection, whether contact or contactless in nature, suggest the possibility of developing early disease prediction from big data [8]. Research on these technologies has been accelerating, particularly that on AI-based approaches, but only a few approaches have been implemented in real clinical settings [9], [10], [11], [12], [13].

Recently, visual contactless technologies have advanced the next level for not only physiological monitoring but also disease detection. A novel contactless video-based technology for rapid screening for carotid artery stenosis, called video motion analysis (VMA), was successfully developed by the authors in 2022 [14]. VMA technology is able to extract useful information from subtle pulses on the neck skin and classify CAS and non-CAS subjects based on the extracted pulse features, with acceptable sensitivity and specificity. The entire screening process, including the capturing of a short video clip from the neck and uploading of the video file to a cloud server for VMA, can be completed without the assistance of medical staff. This advanced technology provides a quick and low-cost option for potential clinical use.

This paper details the development of the PulStroke, a novel, fully-automated screening device for CAS. We decided to take a huge leap and transform the VMA technology into a commercial product by building a user-centric design approach and its support frameworks. An automated system with seamless hardware integration and a streamlined operation flow via backend/frontend software architecture has been developed. We aimed to create a smart CAS screening device with VMA technology that offers an exceptional user experience, meets commercial viability criteria, and complies with regulatory requirements. This device, called the PulStroke as a potential brand name, could be incorporated into future clinical practice to provide the general public with quick and cost-effective CAS screening for the first time.

## 2 Methodology

CAS changes the blood flow patterns in arteries and results in altered pulsation characteristics on the neck skin surface. These subtle but distinct differences in the pulses between healthy and diseased carotid arteries were proven to be detectable in our previous study. The PulStroke project was initiated in an attempt to realize the VMA technology as a commercial product, with the goal of completing the entire CAS screening process in minutes with just one simple screen touch, ensuring a seamless experience for its users.

### 2.1 Background of VMA

VMA involves a series of video processing techniques. Motion magnification is performed on the videos by applying the decomposition of different spatial frequency bands, followed by temporal filtering. The videos are magnified within a specific frequency range, such as the heart rate of a subject, by choosing user-specified parameters for motion magnification. The magnified video is further processed by the optical flow method, allowing for the construction of a flow vector field to highlight the movement of every pixel in each frame. The generated flow vector field is finally processed by principal component analysis to reduce dimensionality and maximize correlations among the variables, resulting in unique pulse information for each subject. The VMA-derived value is then deduced and compared to a pre-determined cutoff line equivalent to 50 % CAS by ultrasound, above which the subject is identified as having a higher CAS risk. In our most recent models, the last part has been further enhanced by using AI technologies instead of principal component analysis to extract more relevant pulse features from videos with more rigorous analysis. A previous clinical study in 2022 achieved an area under the curve (AUC) of 0.914 with 87 % sensitivity and 87 % specificity, validating the feasibility of the VMA technology for non-invasive CAS screening.

### 2.2 Product design

#### 2.2.1 Design goals

**Fig. 1:**
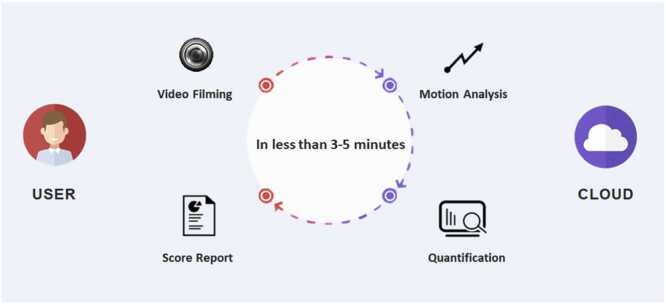
Conceptual flow chart of user experience during screening.

Fig. 1 demonstrates our conceptual flow chart of the user experience during screening. A subject takes a 20-second video clip using the PulStroke device, whether independently or with staff assistance. With just one simple screen touch, the video clip is recorded and automatically uploaded to the Amazon Web Services (AWS) cloud server for the assessment of CAS risk. VMA analyzes the user’s video clip, and a risk report is sent to the user’s registered account in 5 min. The user can check the report on the PulStroke device or on his/her personal mobile device. This summarizes our vision for completing the PulStroke screening process.

#### 2.2.2 Technology Readiness Level

To systematically address this multifaceted task, a structured methodology using Technology Readiness Level (TRL) was developed. TRL is a scale for assessing the maturity of a developing technology or product, and the assessment can inform decisions for further development or commercialization. In the PulStroke project, the initial level of TRL 1 focused on building profiles of users who could benefit from the VMA technology. These profiles included demographic data, vascular health characteristics, and clinical use scenarios. Input was gathered from consultations with specialists, literature review, and feedback from user groups of clinicians and patients. Based on the profiles, TRL 2 proceeded with the creation of design concepts for the PulStroke device, such as its geometric configuration, ergonomic considerations, and desired functional attributes to ensure that clinical and usability needs would be met. At TRL 3 and 4, the device was still under development, with the major emphasis on determining if the individual parts comprising PulStroke would work cohesively. Key tasks included designing the hardware, implementing the VMA algorithms, developing the frontend/backend software architecture, and integrating all the parts together as a system. These tasks were related to our rigorous efforts in the integration of hardware and software. TRL 5 focused on iterative testing and validation of the integrated system. For these purposes, clinical studies were conducted to evaluate the feasibility of the PulStroke and its usability in diverse healthcare settings.

**Fig. 2:**
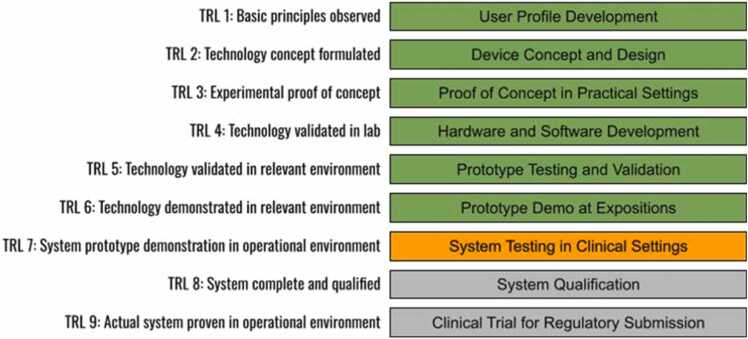
TRL progress in the PulStroke project (completed items shown in green; item in progress, in orange; incomplete items, in gray).

TRL 6 involved the dissemination of the PulStroke device through expositions, medical conferences, and public health campaigns. Extensive efforts have also been made to engage potential stakeholders in discussions about regulatory compliance and commercialization strategies. By adhering to this systematic approach, the TRL status of the PulStroke has climbed steadily from level 1 to level 6, the latter level representing a functional product capable of addressing clinical needs safely and effectively. This systematic approach ensured that all aspects of the PulStroke project were aligned to deliver a cohesive and impactful solution for CAS screening (Fig. 2).

#### 2.2.3 Hardware development

**Fig. 3:**
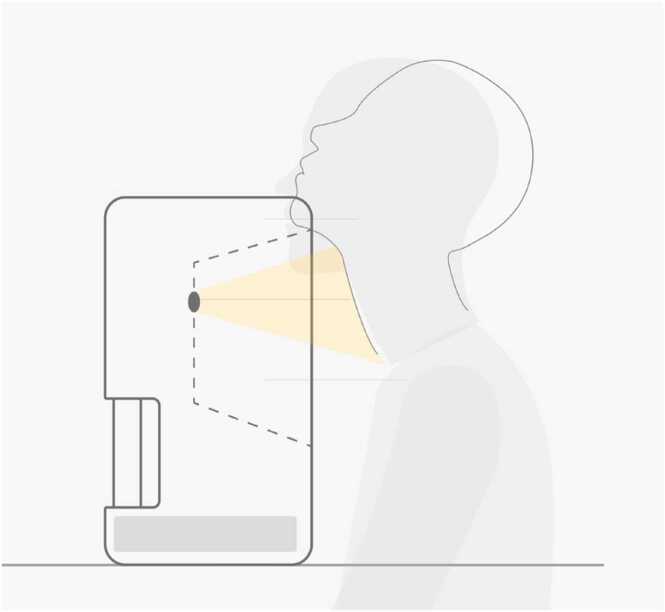
Illustration of video recording setup in sitting position.

In our earlier study, the video clip was recorded with the subject lying in the supine position on a bed, with the head placed inside a custom-made box. This setting, while reducing external factors such as environmental noise or interruptions to the minimum, posed great challenges to commercialization. To improve the convenience of use, the PulStroke device was designed such that the subject would be in a sitting position (Fig. 3). This change in position improved not only the feasibility of the screening but also its efficiency, fostering a more rapid and engaging interaction with its users.

**Fig. 4:**
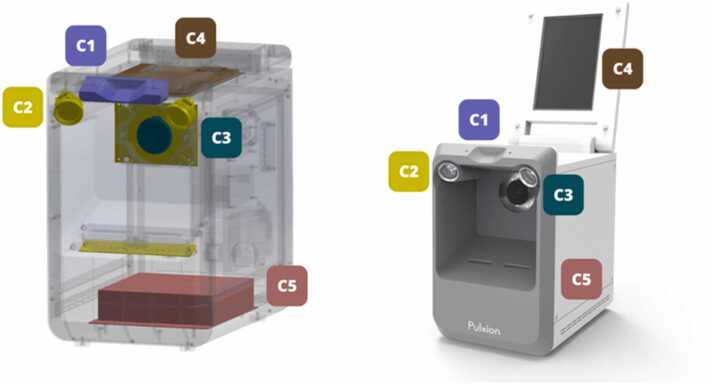
PulStroke hardware design with five major components C1–C5.

The PulStroke hardware consists of five major components: an illumination system, an optical camera, a flip-top touch-screen display panel, a mini-computer, and a metal–plastic enclosure housing the components (Fig. 4). The metal–plastic enclosure has the geometric dimensions of a typical desktop computer tower, being long enough to provide the depth of field for the optical camera to focus and tall enough to allow for neck stretching by the subjects. A specialized notched chin rest (C1) helps hold the subject’s chin, and by extension the neck, in place for video recording. The illumination system (C2), controlled through a printed circuit board, has a circular front light surrounding the optical camera (C3), a pair of side lights projecting from the top at a 45° angle, and a bar light at the bottom. The subject's neck is thus illuminated to enhance the video quality, ensuring optimal visibility of the carotid artery pulses. A customized touch screen (C4) is embedded in a metal–plastic flip-top display panel to guide the users throughout the screening process. A mini-computer (C5) provides a local platform for communications among the hardware, software, and cloud server.

#### 2.2.4 Software architecture development

**Fig. 5:**
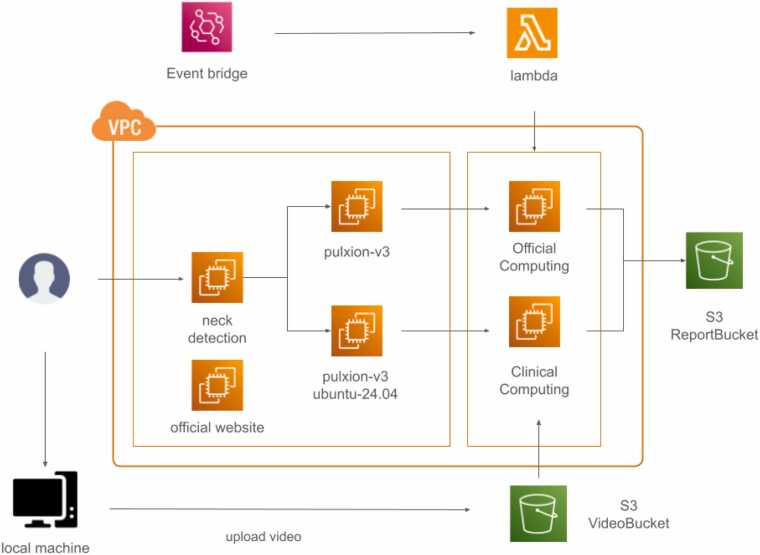
PulStroke software architecture in the AWS environment.

The software workflow shown in Fig. 5 was built on AWS within a secure Virtual Private Cloud (VPC) environment. This architecture provides a logically isolated section within the AWS cloud, giving developers full control over the virtual network, IP address ranges, subnets, and security configurations. The scalable cloud computing services (AWS EC2) and object storage solutions (AWS S3) are used to manage the workflow efficiently. When a user uploads a video from a PulStroke device, it is sent to a secure storage bucket, which triggers an automated processing sequence. The video first undergoes neck detection to define a region of interest (ROI), followed by VMA. The risk assessment reports are stored in a separate and secure bucket, accessible via the PulStroke user interface. This system architecture was designed to be compliant with international data privacy regulations, such as HIPAA and GDPR, ensuring patient confidentiality through robust de-identification and cryptography for all patient data before video uploading to the cloud server. A detailed description of the software architecture can be found in the supplementary information.

### 2.3 Usability test

The usability of the PulStroke device was evaluated to ensure it met the usability and accessibility needs of its intended users of different ages and occupations. Thirty medical and non-medical professionals were recruited as participants to reflect the wide diversity of the intended users. These participants were divided into five distinct age groups (in years): 20–29 (11), 30–39 (4), 40–49 (8), 50–59 (5), and 60–69 (2). All participants consented to the provision of post-screening feedback, and their data were anonymized for analysis.

**Fig. 6:**
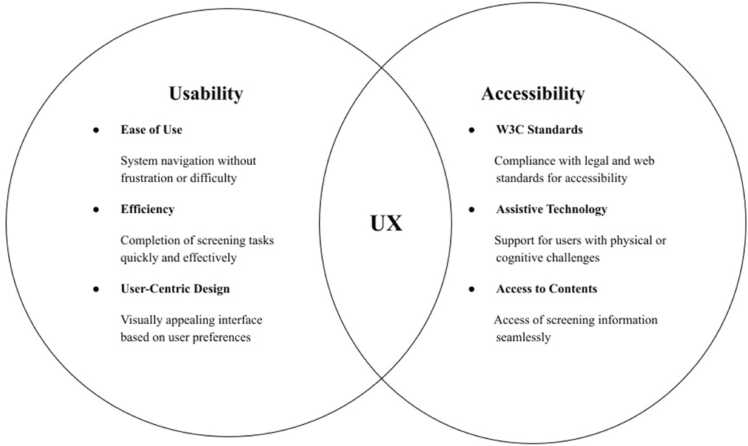
PulStroke user experience evaluated by usability and accessibility.

Thirteen of the participants were enrolled in the usability test, with an observer sitting next to them to evaluate their task performance. The usability test was conducted by first observing their task performance and then collecting feedback on their user experiences. These participants completed tasks with or without reading the Instructions for Use (IFU) while being observed. Quantitative metrics (complete and incomplete rates, and qualitative feedback collected through post-screening surveys) were analyzed to identify the strengths and weaknesses in the system’s usability. This test provided a comprehensive framework to assess the usability and accessibility of the PulStroke device, ensuring its design aligned well with user needs and expectations (Fig. 6).

## 3 Results and Discussion

We aimed to create a CAS screening device with VMA technology, i.e., the PulStroke, that would offer an exceptional user experience. An automated system integrating hardware and software architecture was developed. The PulStroke project was initiated to turn the VMA technology into a viable consumer product, with the goal of completing CAS screening quickly and with ease to ensure a seamless user experience.

### 3.1 Hardware development

#### 3.1.1 PulStroke evolution

**Fig. 7:**
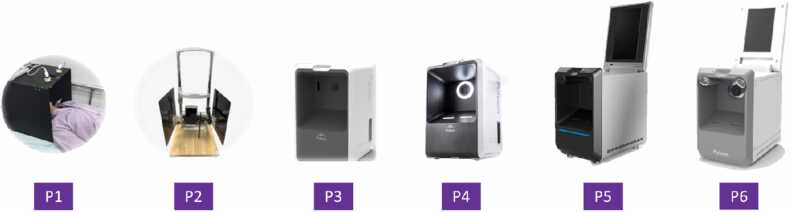
PulStroke hardware evolution from TRL 1 to TRL 6.

The hardware development of the PulStroke device was in general aligned with the TRL framework illustrated in Fig. 2, progressing systematically through six levels (Fig. 7). At TRL 1, the VMA principles were conceptualized and validated through testing in a well-controlled lab environment, with the subject lying in a supine position. Transitioning to TRL 2, a device in the sitting position was explored for the first time, making it feasible in a practical scenario. At TRL 3, a preliminary prototype with a sleek look was fabricated using 3D printing and manually assembled, allowing for iterative refinements of its ergonomic performance and functional attributes. At TRL 4, an enhanced version of the device was developed by integrating an auxiliary illumination system to improve the video quality in diverse environments.

TRL 5 focused on adopting a flip-top touch-screen display panel which was integrated into the device, allowing a user to perform the screening without any assistance. The culmination of all efforts came at TRL 6, where the hardware was fabricated precisely using Computer Numerical Control (CNC) technology, with modular designs incorporated to enhance its functionality and scalability for mass production. This stage included extensive testing and validation in diverse healthcare environments, demonstrating the readiness of the device for real-world deployment. Through this systematic approach, the PulStroke hardware advanced from conceptualization to a fully operational product, ensuring alignment with clinical and usability needs. This user-centered design and ergonomic layout, comprising the five major components illustrated in Fig. 4, were seamlessly integrated in the hardware infrastructure. Table 1 summarizes the evolution of the PulStroke hardware from TRL 1 to TRL 6.

**Table 1:** PulStroke hardware evolution from TRL 1 to TRL 6.

**HW Design**	**Technology Readiness Level**	**Position**	**Characteristics**
P1	TRL 1	Supine	Subject lying on bed
P2	TRL 2	Sitting	Subject sitting
P3	TRL 3	Sitting	3D printed prototype
P4	TRL 4	Sitting	Auxiliary lighting system added
P5	TRL 5	Sitting	Flip-top touch-screen panel added
P6	TRL 6	Sitting	CNC with modular design for production

After the completion of TRL 6, the PulStroke device was sent to a third-party inspection agency to prove its conformity to safety and performance standards for future regulatory submission. These tests included electromagnetic compatibility, electrical safety, biocompatibility, photobiological safety, and environmental testing. The PulStroke device passed all required tests and was awarded certificates validating its safety and performance.

#### 3.1.2 System Integration

**Fig. 8:**
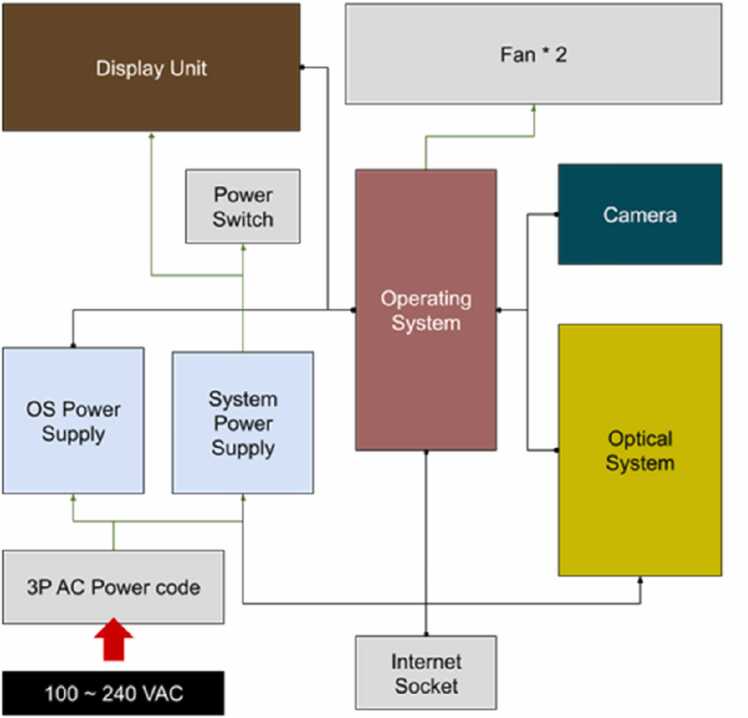
System integration of hardware components.

To ensure operating functions such as communication and power management, the hardware infrastructure is seamlessly integrated, as shown in Fig. 8. The operating system (OS) in the mini-computer serves as the core controller, managing communications among the major components such as the optical camera and illumination system. Once the screen panel is touched and commands are received, the OS is activated to complete two actions: (1) the optical camera captures high-resolution videos for VMA, and (2) the illumination system provides appropriate lighting to enhance the video quality. The dots in Fig. 8 represent the information communications among dotted components.

When the screening process is completed, the internet socket, which provides a stable internet connection, facilitates data transmission for the cloud-based VMA. Two cooling fans maintain the thermal stability inside the device to prevent overheating during screening. The entire system is powered by a 100–240 VAC input through a 3 P AC power cord, and the electricity is distributed by an OS power supply and system power supply. The former supplies the OS to ensure reliable operation, while the latter supplies the components, such as the display unit and camera. The directions of the arrows in Fig. 8 indicate the current flows to the supplied targets.

### 3.2 Software architecture

#### 3.2.1 Backend

**Fig. 9:**
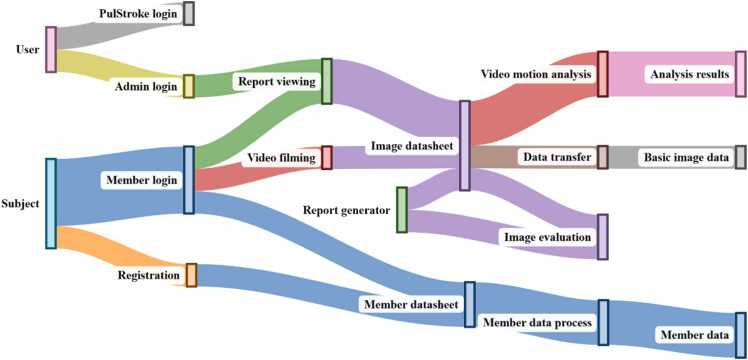
Screening workflow from user/subject to various processes.

The screening workflow of the PulStroke device shown in Fig. 9 integrates the user and subject interactions with streamlined video processing and report generation to support clinical standards and usability needs. Here, a subject is an individual participating in studies designed to assess the accuracy, reliability, and performance of the PulStroke device. These participants include both healthy individuals and patients with symptomatic and asymptomatic CAS. In contrast, a user is usually an administrator, with or without a medical background, who assists subjects during screening or retrieves reports from local devices, if needed. It should be noted that the PulStroke device can also be operated independently by a subject without any assistance, with the option of receiving the screening report on the subject’s personal mobile device.

A user begins the process by accessing the PulStroke device through its login interface, enabling tasks such as viewing reports on local devices or initiating the VMA on the cloud servers. A subject logs in via the member login portal, where he/she can either register to generate member-related data or proceed with video capture. The video clip flows into the image datasheet, which centralizes the processing tasks, including data transfer, to generate basic data and VMA for assessing CAS risk. The latter leads to report generation, which produces a finalized screening report indicating low or high risk. This report is accessible from a local PulStroke device or a personal mobile device. Simultaneously, member-related data are organized and sent to the member datasheet. This approach ensures an integrated workflow for collecting, analyzing, and delivering CAS screening efficiently.

#### 3.2.2 Frontend

**Fig. 10:**
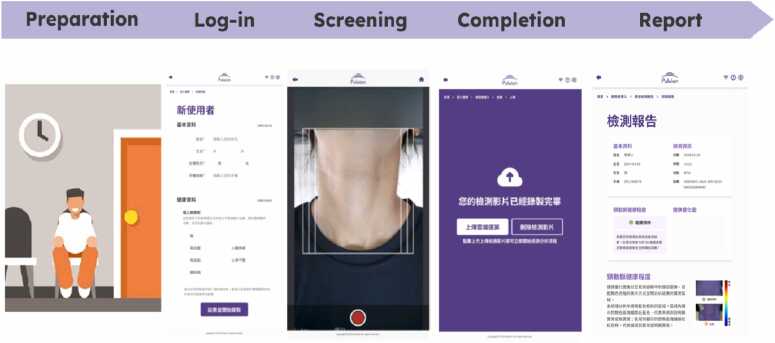
Systematic screening process in five major steps.

The PulStroke CAS screening process, supported by the frontend user interface, includes five major steps: preparation, login, screening, completion, and reporting (Fig. 10). Preparation involves checking the device, ensuring a clean background, and advising subjects to keep the neck area exposed for the camera. To ensure that the neck is placed in a correct posture, subjects are reminded to put their chins on the notched chin rest (C1 in Fig. 4).

In the login phase, a user logs into the system, enters the subject's information, and ensures device connectivity. The touch screen (C4) on the flip-top display panel offers the data input function and serves as the major host for the graphical user interface. Data can be input via a keyboard built into the panel for user convenience. When the user hits the bottom of the touch screen to begin recording, the illumination system (C2) and optical camera (C3) are turned on automatically to record a 20-sec video. The mini-computer (C5) converts the video into a storage file and uploads it to the cloud server for VMA. Within five minutes, the system completes the VMA and generates a screening report, which is sent back to the local PulStroke device and the subject’s registered account, accessible by personal mobile device. This systematic workflow, as shown in Fig. 10, is supported by major hardware components (C1–C5) and streamlined by AWS IoT integration (Fig. 5) to ensure an efficient screening process.

### 3.3 Screening demonstration

To demonstrate the functionality of the finalized TRL 6 prototype, five cases with various degrees of CAS were presented. It should be noted that these cases were intended to highlight the capability of the PulStroke to assess CAS risk, not to serve as a clinical validation of its screening accuracy. A large-scale, multi-center clinical trial is currently underway to validate the clinical performance of the PulStroke.

**Fig. 11:**
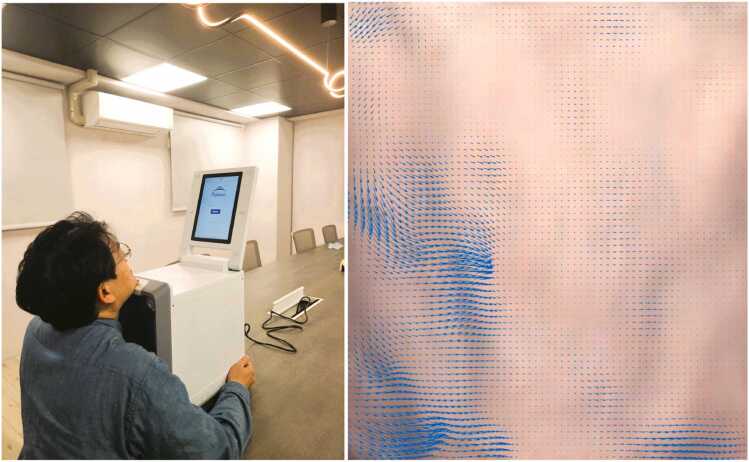
PulStroke setup (left); Movements of every pixel in each frame (right).

Subjects sat in front of the PulStroke device with the neck stretched as shown in Fig. 11. The video clip was recorded for 20 s at 30 frames per second and a pixel resolution of 1920 × 1080. A rectangular ROI, focused below the subject’s chin and above the subject’s clothing, was automatically boxed by AI object detection for the subsequent VMA. The VMA involved a series of video processing techniques such as motion magnification and optical flow. The optical flow determined the pulse motion of the subjects between consecutive frames and allowed for the construction of a flow vector field, highlighting the movement of every pixel in each frame (Fig. 11). AI technologies were used to extract relevant pulse features from videos using rigorous regression models for accessing CAS risk.

**Fig. 12:**
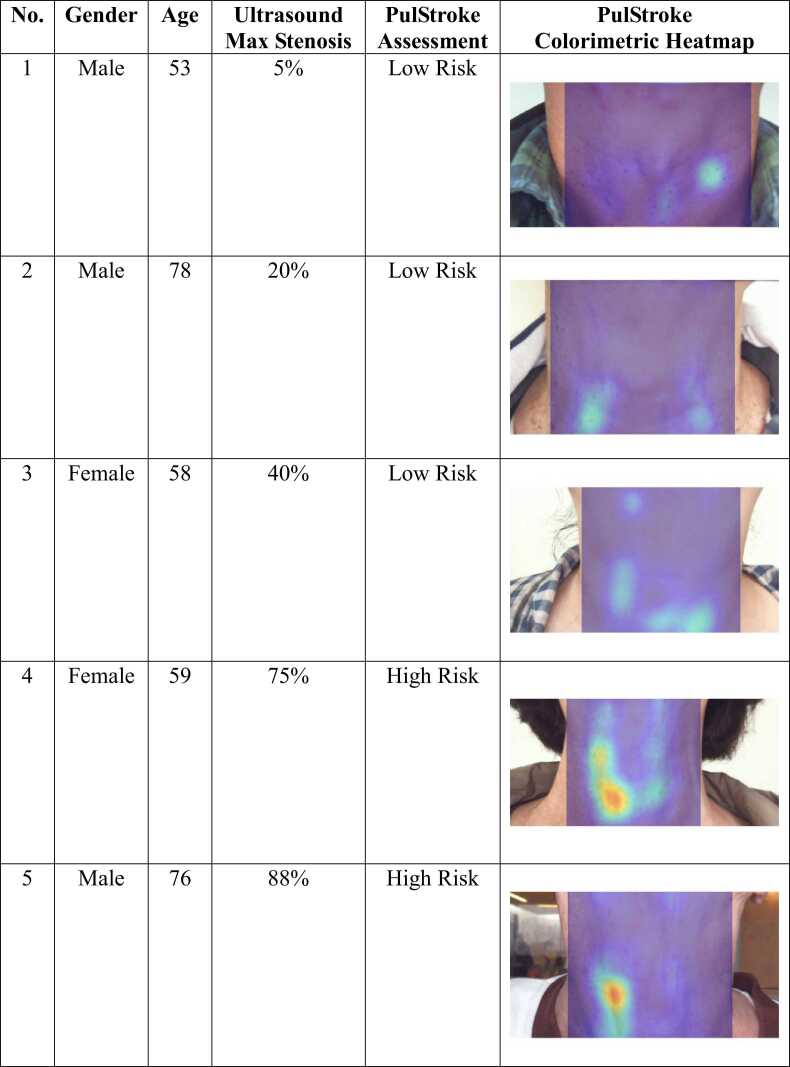
Colorimetric heatmaps indicating low to high CAS risk.

After the VMA, three subjects were identified as low-risk and the other two as high-risk. The PulStroke generates colorimetric heatmaps to visually present its screening results and findings. In a colorimetric heatmap, different colors or shades of color are used to indicate different risk levels. In our heatmap, a gradient from blue to red is used, where blue represents lower risk and red represents higher risk. This allows users to quickly identify patterns and trends for CAS risk. The top three images in Fig. 12, corresponding to the three low-risk subjects, show less heat intensity, while the bottom two images, representing the two high-risk subjects, display pronounced localized heat intensity with the color red. These five subjects were subsequently examined by carotid Doppler ultrasound, and the latter two were proven positive with maximum stenosis of 75 % and 88 %, respectively. This outcome demonstrates the effectiveness of the PulStroke device in distinguishing CAS risk levels. This screening process will be further validated in an upcoming multi-center clinical trial for regulation submission in the near future.

### 3.4 Usability test

Fifteen medical professionals and fifteen non-medical professionals consented to the provision of post-screening feedback. Thirteen of them were enrolled in the usability test and consented to taking the user task performance survey anonymously, with an observer sitting next to them to evaluate their task performance. All participants were provided with Instructions for Use (IFU) but were free to choose whether or not to read them. The participant information is summarized in Table 2.

**Table 2:** Participant information: Post-test feedback.

**Information**	**Number**	**Percentage (%)**
**Male**	12	40.0
**Female**	18	60.0
**Age group (years)**		
**20–29**	11	36.7
**30–39**	4	13.3
**40–49**	8	26.7
**50–59**	5	16.7
**60–70**	2	6.6
**Medical Professionals**	15	50.0
**Non-medical Professionals**	15	50.0

At this stage, the operation requires the presence of an assistant (medical or non-medical) to ensure smooth execution and support the subject in completing the process. However, in the next phase, we plan to conduct usability tests aimed at empowering the subject to operate the system independently, without any assistance, including more diverse populations such as elderly and non-technical users. Ultimately, our goal is to transition from assisted operation to a streamlined and self-directed experience accessible to the general public.

#### 3.4.1 User task performance

To evaluate the participant performance in completing tasks, quantitative metrics such as completion rate (CR) and standard deviation (SD) were calculated, as shown in Table 3. To quantify how well the subjects were prepared for screening, participants were evaluated to see if they followed the instructions, with or without referring to the IFU. If the participant completed a step correctly, a score of 1 was given; otherwise, a score of 0 was recorded. A total of seven user tasks were evaluated, as follows: (1) subject preparation, (2) user login, (3) device setup, (4) new account registration, (5) video recording and analysis, (6) report access, and (7) data storage. Each task shown in Table 3 plays a crucial role in maintaining the procedural integrity.

**Table 3:** Completion rate for each task in user task performance.

**Task**	**Aim**	**CR (SD)**
**Task 1: Subject Preparation** The participant prepared the subject for screening by seating them on a chair, with the chin placed on the chin rest and the neck stretched.	The subject was informed of the screening procedure. The chin was positioned on the chin rest to ensure stability during screening. Chin rest isolation paper had to be replaced for each subject.	0.46 (0.52)
**Task 2: User Login** The participant logged into the PulStroke device with a pre-established account via touch screen panel.	This step ensured traceability of the data and results, in compliance with data security protocols.	0.92 (0.28)
**Task 3: Device Setup** The participant ensured that the device was functioning properly, with the background clean and cleared.	The background should be clean, avoiding excessive lighting, shadows, or moving objects that could interfere with the video quality.	0.87 (0.33)
**Task 4: New Account Registration** The participant entered the subject’s information into the system via touch screen panel.	Subject basic information, such as name and consent to data collection, was entered into the system.	1.00 (0.00)
**Task 5: Video Recording and Analysis** The participant initiated the screening process of recording and VMA by following the instructions displayed on the touch screen.	The video was taken and processed by VMA, which ran a trained predictive model to assess the CAS risk.	0.96 (0.20)
**Task 6: Report Access** Once the VMA was completed, screening results could be accessed from the device for review by the participant.	The participant assessed the screening reports, confirming the CAS risk of the subjects.	0.92 (0.28)
**Task 7: Data Storage** Data and results were saved and securely stored in the system for future reference and reporting.	All subjects’ personal data and screening results were automatically archived.	0.92 (0.28)

The placement of the chin rest isolation paper was the major action item to be evaluated in Task 1, “Subject Preparation”. The completion rate was low, 0.46 with an SD of 0.52, indicating that less than half of the participants completed this task correctly. Three of the participants did not read the IFU, and none of these three completed this task. This outcome showed that reading the IFU was highly correlated with user task performance and thus will be requested in the future. In Task 3, “Device Setup”, the completion rate was defined by three criteria: device powered on, power cord connected, and network connected. Additionally, the background should be clean, i.e., free of improper lighting, shadows, or moving objects that could interfere with the video quality. The final completion rate was 0.87 with an SD of 0.33, the second lowest next to Task 1, reflecting that some participants were unaware of noise issues in the surrounding environment.

Completion of Task 4, “New Account Registrations”, was defined as successful registration for a new account by the subject. Its completion rate was 1.00, also the highest among all seven user tasks. The remaining four user tasks had completion rates higher than 0.90, indicating that the majority of the participants were able to perform these tasks with high success rates and minimal variability.

#### 3.4.2 Satisfaction feedback

To assess user satisfaction, qualitative feedback was collected from thirty medical and non-medical professionals using a questionnaire. For this assessment, each question was scored on a 5-level scale of 0 (disagreement) to 4 (agreement) to evaluate the degree of user satisfaction. The tallied results from all participants were then normalized to 0–1, with 1 being the most satisfactory. The two most representative questions from the questionnaire are listed in Table 4 for further discussion. To define the success of the PulStroke development, the question “The PulStroke functions are complete and easy to use” was asked. It was rated as high as 0.98 (98 % satisfaction) and no lower than 0.88 by different groups. This suggests that most of the participants found the PulStroke to be fully functional and easy to use due to our user-centric design approach. Interestingly, participants who did not read the IFU rated the system’s ease of use slightly higher than those who did. This may demonstrate that the PulStroke user interface is highly intuitive, allowing users to naturally understand how to operate it through exploration. On the other hand, participants who read the IFU may have tried to follow the IFU step-by-step, even when the interface suggested a more direct or obvious path. Therefore, a future improvement will be to integrate key instructions directly into the on-screen interface, making them an essential part of the screening process to minimize operator error, particularly for first-time users.

**Table 4:** Survey questions and outcomes for satisfaction evaluation of participants.

	**Medical Professionals**		**Non-Medical Professionals**	
	**IFU** **(n = 4)**	**Without IFU** **(n = 11)**	**IFU** **(n = 10)**	**Without IFU** **(n = 5)**
The PulStroke functions are complete and easy to use.	0.88 (0.58)	0.98 (0.30)	0.90 (0.52)	0.95 (0.45)
The PulStroke requires professional assistance when first using the device.	0.25 (1.50)	0.18 (1.01)	0.63 (1.08)	0.60 (1.82)

To better understand the necessity of human intervention in the scenario of the first-time user, another question, “The PulStroke requires professional assistance when first using the device”, was also evaluated. Here, the medical professionals showed that they were highly independent with (0.25) or without (0.18) reading the IFU, while the non-medical professionals showed moderate dependency, with around 0.60 requiring assistance their first time using the device. The difference between the medical and non-medical professionals was expected due to the fact that medical professionals tend to be very experienced in handling various medical devices, making additional assistance likely to be unnecessary. Conversely, more than half of the non-medical professionals, lacking routine training with such devices, required assistance for their first time.

Given the fact that most users did not proactively read the IFU, an important future improvement will be to integrate the IFU instructions directly into the user interface. This would require users to review the necessary steps before proceeding with the screening process to reduce operational errors, particularly for users without a medical background.

### 3.5 Future directions

The PulStroke has successfully reached TRL 6, signifying that a fully-functional prototype has been demonstrated in a relevant environment. To advance toward TRLs 7 through 9, the following steps are currently in progress:

Building a Quality System: We are in the process of establishing a robust quality management system (QMS) in compliance with international regulatory standards. These include ISO 13485 for medical devices quality management, IEC 60601 for electrical safety and performance, and IEC 62304 for the software development life cycle. This framework will ensure consistent product development, manufacturing, quality, and documentation to address key requirements for product safety and effectiveness.

**Fig. 13:**
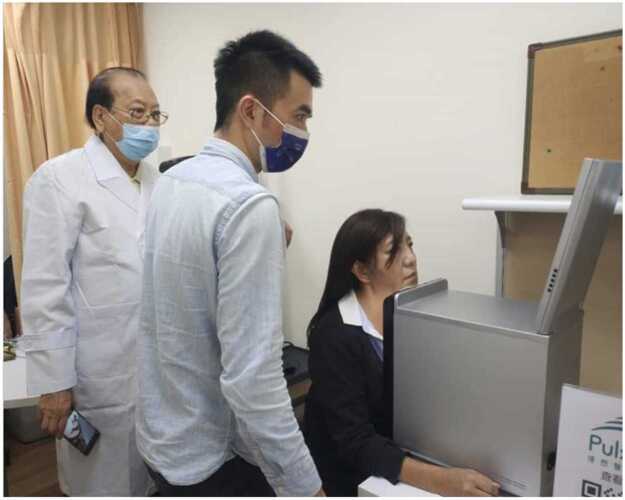
PulStroke demonstration in real-world clinical setting.

Preparing for a Clinical Trial: We are preparing for a multi-center clinical trial to validate the PulStroke performance in a real-world setting (Fig. 13). This trial is designed to establish the screening accuracy, sensitivity, and specificity of the PulStroke device by comparing its screening results against the clinical gold standard, carotid Doppler ultrasound, across a large and diverse patient pool. Three medical centers have been chosen, and the IRB evaluation is currently in progress. This clinical trial will provide the necessary data and information for final validation.

Expanding Usability Testing: To validate the usability and accessibility of the PulStroke, we conducted an initial usability test with a smaller sample size (N = 30), which yielded encouraging data and confirmed the system’s usability and accessibility. Recognizing the importance of comprehensive user feedback, we have launched an ongoing usability testing campaign to expand our dataset and refine the user experience. Although accumulating sufficient data takes time, this methodical approach reflects our commitment to future product success. In addition, we plan to conduct usability tests aimed at empowering the subject to operate the system independently, without any assistance, including more diverse populations such as elderly and non-technical users.

**Fig. 14:**
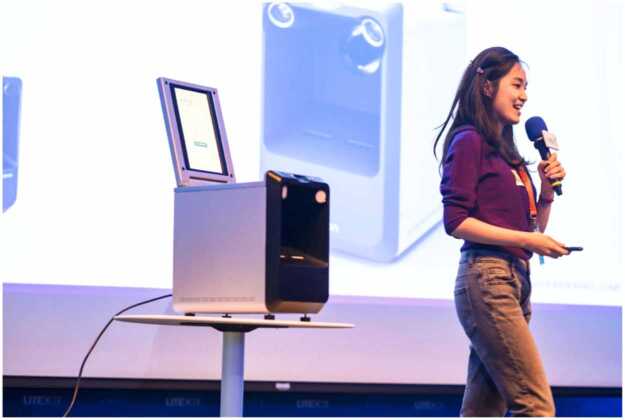
PulStroke demonstration on stage at exhibition.

Showcasing at Key Exhibitions: We have participated in many major exhibitions (e.g., MEDICA) and medical conferences to give PulStroke demonstrations (Fig. 14) to highlight the features and potential impact of the device, attracting medical professionals and early adopters while gathering valuable market feedback.

These actions aim to push the PulStroke to TRL 9, which signifies that a fully-operational product is ready for market launch and global use. This is an engaging journey toward realizing an innovative concept as a commercial product, which is especially challenging in an academic environment.

This paper is intended to demonstrate the technical capability of PulStroke to assess CAS risk, not to serve as a clinical validation of its screening accuracy. Our current focus is to establish device feasibility and functionality, laying the groundwork for subsequent clinical validation. The five clinical cases presented are preliminary examples that showcase the potential of the device and its feasible integration into clinical practice. While we acknowledge the importance of broader clinical validation, such efforts are beyond the scope of the current work and are planned for the upcoming large-scale, multi-center clinical trial.

## 4 Conclusions

The PulStroke is the world’s first fully-automated screening device for carotid artery stenosis. This paper presents the evolution of the PulStroke device from concept to product, the technology readiness level of which has advanced from 1 to 6. With the PulStroke, a user records a 20-second video clip, and with just one simple screen touch, the video clip is automatically uploaded to the cloud server for the risk assessment of CAS in 5 min. It is an automated system with hardware integration and a streamlined operation flow via backend/frontend software architecture. The path forward for realizing this purpose presented great challenges when the project was initiated, especially in an academic environment. After years of persistence and innovation, what was once an initial concept has now become a tangible product.

Target users were invited to participate in the PulStroke usability testing. This was crucial in assessing the device's safety, effectiveness, and practicality in real-world settings. The feedback was very positive, with more than 88 % of the participants expressing satisfaction with the device. This strong approval indicates its user-friendly design and potential to make an impact in the medical field. PulStroke screening outcomes will allow early interventions for millions of people, reducing healthcare costs and improving survival rates around the globe.

## CRediT authorship contribution statement

**Li-Han Lin:** Formal analysis, Methodology, Validation, Visualization, Data curation, Investigation, Supervision, Writing – original draft. **Ching-Chang Huang:** Data curation, Investigation, Resources, Validation, Formal analysis, Project administration, Supervision. **Yu-Ting Shen:** Investigation, Data curation, Methodology, Validation, Project administration. **Ko-Wei Fan:** Data curation, Software, Formal analysis, Validation. **Hsien-Li Kao:** Investigation, Supervision, Writing – review & editing, Funding acquisition, Project administration, Conceptualization, Resources, Visualization. **Hsin-Fang Hsu:** Supervision, Investigation, Validation, Data curation, Methodology. **Peng-Jie Wang:** Formal analysis, Software, Visualization, Data curation, Methodology, Validation, Investigation. **Hao-Ming Hsiao:** Conceptualization, Data curation, Formal analysis, Investigation, Funding acquisition, Methodology, Project administration, Resources, Software, Supervision, Validation, Visualization, Writing – original draft, Writing – review & editing.

## Declaration of Competing Interest

The authors declare the following financial interests/personal relationships which may be considered as potential competing interests: Drs Hsiao and Kao cofounded a startup company, Pulxion Medical Technology, in 2020 to translate their research work into clinical solutions. The remaining authors declare that they have no conflicts of interest.
