# Supplementary material for: Novel rapid screening device for carotid artery stenosis using video motion analysis: From concept to product
Source: Comput Struct Biotechnol J. 2025 Oct 30;28:468–79. doi: 10.1016/j.csbj.2025.10.055 (PMC12603725; doi:10.1016/j.csbj.2025.10.055)
Supplement: Supplementary file 1 — Supplementary material [file mmc1.doc]

**Supplement**

Software Architecture Development

The software workflow utilizes the AWS service for VMA in a secure Virtual Private Cloud (VPC) environment. The VPC is designed to facilitate the provision of a logically isolated section within the AWS cloud, where its resources can be deployed in a user-defined virtual network. Total control over this virtual networking environment is granted to the developers, including the selection of IP address ranges, creation of subnets, and configuration of route tables and network settings.

A web service, Elastic Compute Cloud (EC2), offers a scalable computing capacity within the cloud environment. Total control over the computing environment is granted to the developers as well, enabling users to tailor resources to their specific needs. The time required to launch new servers is significantly reduced to merely minutes, facilitating rapid upward or downward scaling of computing capacity based on evolving computational requirements in real time.

The videos are captured and uploaded by the users from their local PulStroke devices to the cloud (S3 VideoBucket, to be specific), triggering an EventBridge rule that invokes a Lambda function to initiate video processing. A simple storage service, S3, is an object storage solution designed to deliver data, security, and performance. A simplified web service interface is provided to facilitate the storage and retrieval of data in any volume, at any time, and from any location on the web. Data are stored in containers referred to as S3 buckets, which function as the repositories for objects. This system is built upon a highly reliable, secure, and cost-efficient infrastructure identical to that employed by Amazon to support its global network.

Once videos are uploaded, they first go through neck detection to box a region of interest (ROI), followed by VMA using the Pulxion-v3 version, with an additional Ubuntu 24.04 version for handling specific cases. These two versions are processed through two different workflows, with “Official Computing” for standard analysis and “Clinical Computing” for specific cases. The assessment reports generated by EC2 are stored in an S3 ReportBucket, where they are accessible via the PulStroke website for review. Our software architecture efficiently manages the user interface, video uploading, VMA, and report generation with seamless integration of several cloud services. This workflow also ensures the confidentiality of sensitive health data by de-identification and cryptography processing to protect personal information before uploading of the video.
